# Supplementary material for: MD2 activation by direct AGE interaction drives inflammatory diabetic cardiomyopathy
Source: Nat Commun. 2020 May 1;11:2148. doi: 10.1038/s41467-020-15978-3 (PMC7195432; doi:10.1038/s41467-020-15978-3)
Supplement: Supplementary file 1 — Supplementary Information [file 41467_2020_15978_MOESM1_ESM.pdf]

Supplementary Information

MD2 activation by direct AGE interaction drives inflammatory diabetic cardiomyopathy

Wang *et al.*

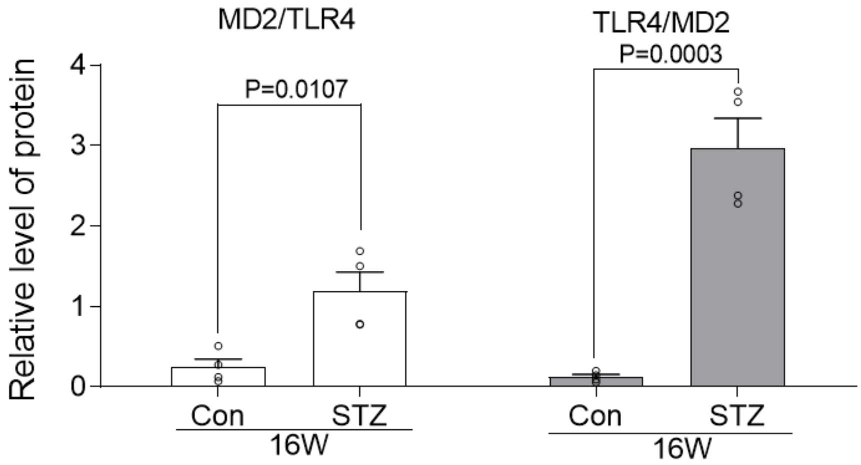

Supplementary Figure 1: MD2-TLR4 interaction in heart tissues of diabetic mice. Densitometric quantification of blots in Figure 1b. Values are reported as Mean ± SEM [n = 4]. P-values by unpaired t test are indicated.

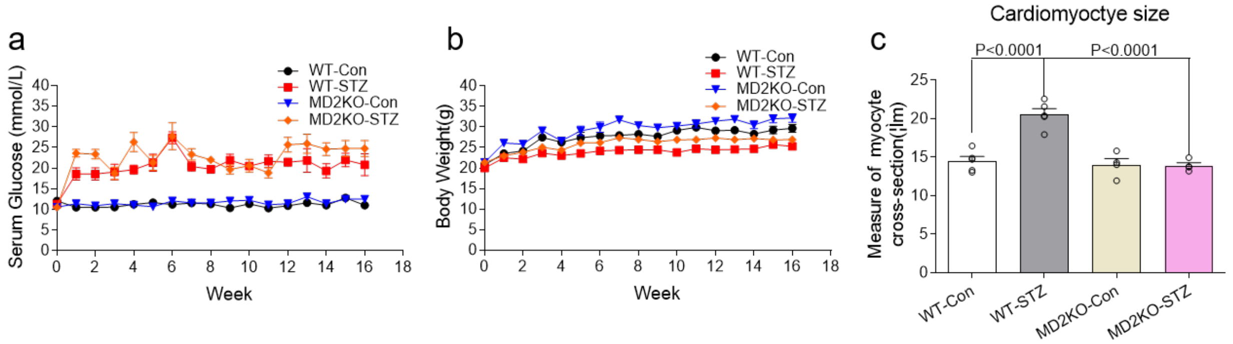

Supplementary Figure 2: MD2 knockout mice are protected against diabetes-induced cardiomyocyte hypertrophy.

(**a**, **b**) Diabetes was induced in mice by streptozotocin (STZ). Fasting blood glucose levels (a) and body weights (b) were measured weekly for 16 weeks [WT-Con = nondiabetic controls, n=6; WT-STZ = STZ-induced diabetic mice, n=7; MD2KO-Con = nondiabetic MD2$^{-/-}$ mice; n=6; MD2KO-STZ, STZ-induced diabetic MD2$^{-/-}$ mice, n=7; Values are reported as Mean ± SEM]. (**c**) Heart tissues were harvested from mice at week 16 and stained with H&E to measure cardiomyocyte size in cross-section areas. Quantitative data of myocyte cross-section length of 100 cells randomly selected in different microscopic fields is shown (n=5 per group). P-values in **c** by one-way ANOVA followed by Tukey's post hoc test are indicated.

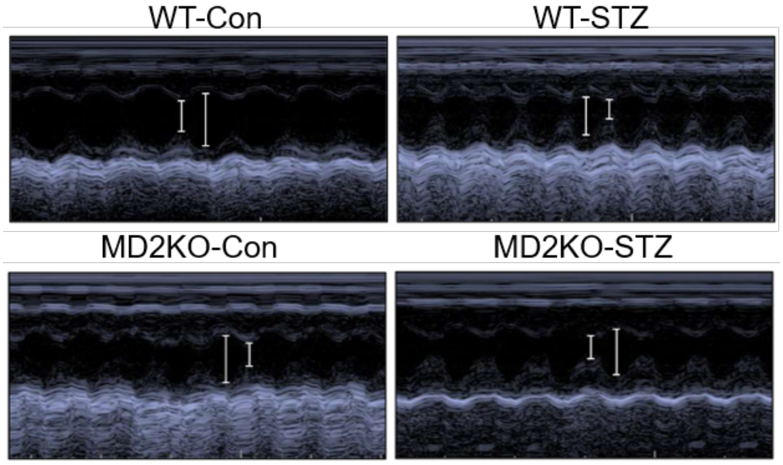

Supplementary Figure 3: Representative left ventricular M-mode echocardiographic tracings at 16 weeks. [WT-Con = nondiabetic controls, n=6; WT-STZ = STZ-induced diabetic mice, n=7; MD2KO-Con = nondiabetic MD2-/- mice, n=6; MD2KO-STZ, STZ-induced diabetic MD2-/- mice, n=7].

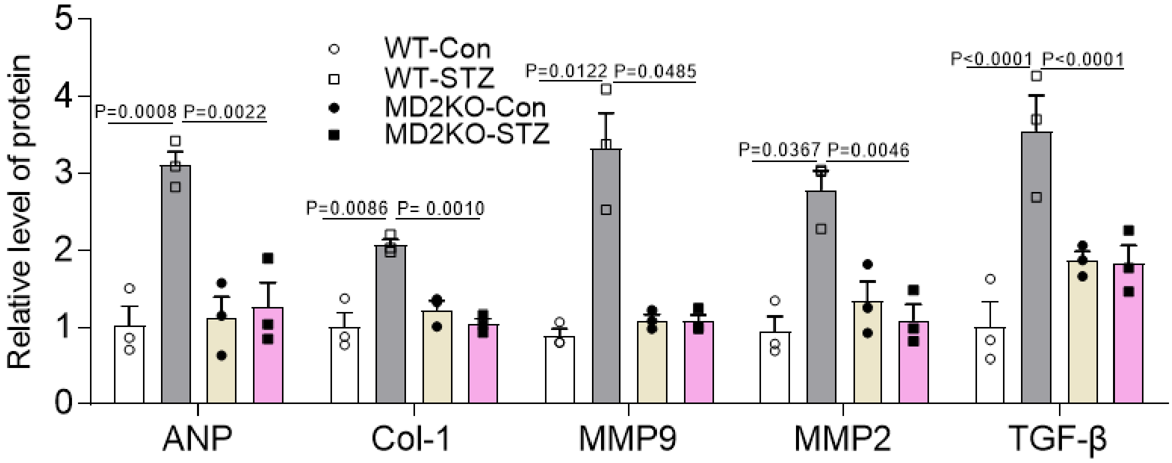

Supplementary Figure 4: MD2 deficiency prevents diabetes-induced cardiac remodeling genes. Densitometric quantification of indicated blots (compared to GAPDH), in Figure 2c. Values are reported as Mean ± SEM [n = 3 per group]. P-values by one-way ANOVA followed by Tukey's post hoc test are indicated

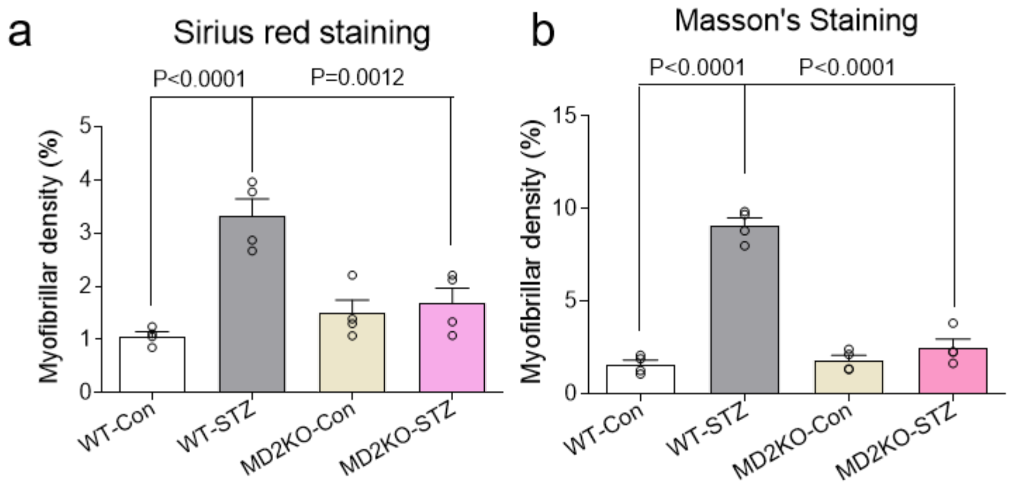

Supplementary Figure 5: Morphological alterations in heart tissues of diabetic mice. Quantification of Sirius Red (**a**) and Masson's Trichrome (**b**) is shown. Representative staining images are presented in Figures 2d and 2e. Values are reported as Mean ± SEM [n = 4]. P-values by one-way ANOVA in **a, b** followed by Tukey's post hoc test are indicated.

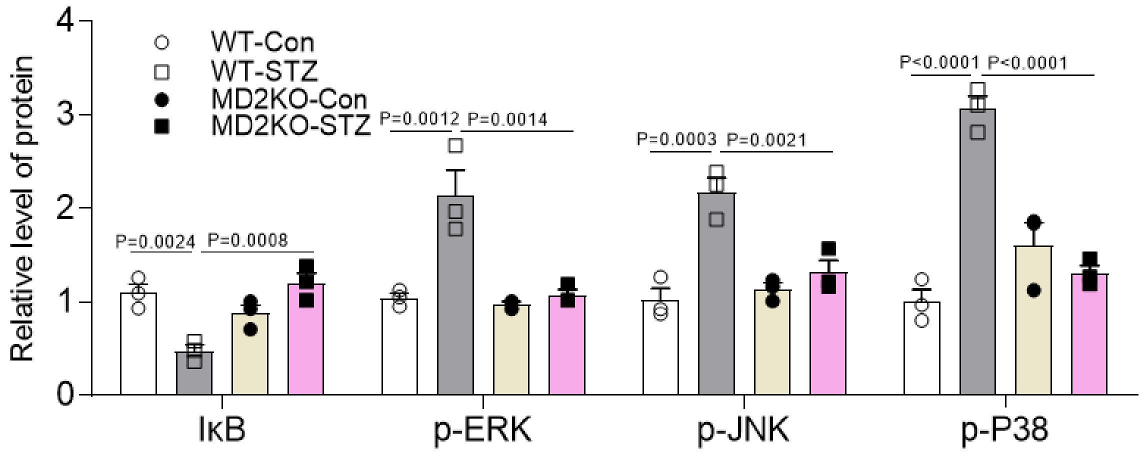

Supplementary Figure 6: NF-κB and MAPK activation in heart tissues of diabetic mice. Densitometric quantification of blots (compared to GAPDH) is shown. Representative immunoblots are presented in Figure 2h. Values are reported as Mean ± SEM [n = 3]. P-values by one-way ANOVA followed by Tukey's post hoc test are indicated.

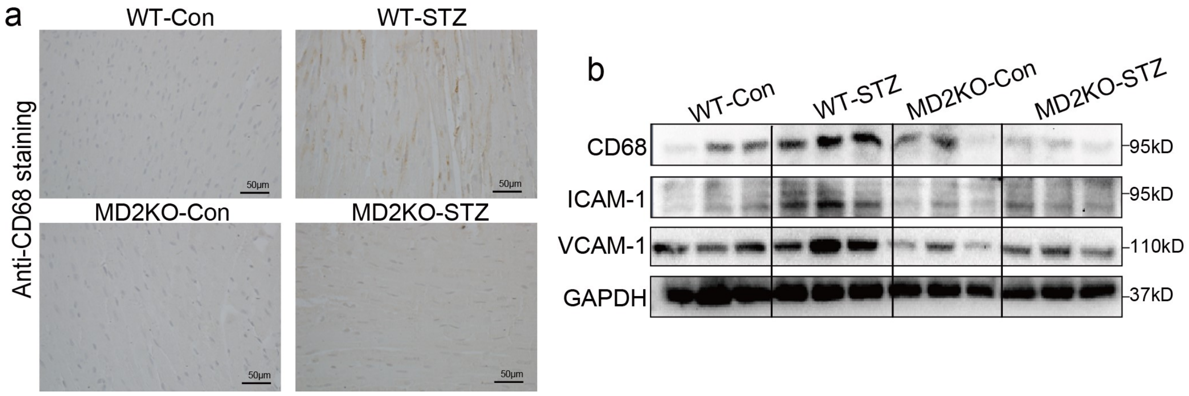

Supplementary Figure 7: MD2 deficiency prevents macrophage infiltration in hearts of diabetic mice. (**a**) Representative immunohistochemcal staining of heart tissues for macrophage marker CD68. Heart tissues were harvested from mice at 16 weeks following the onset of streptozotocin (STZ)-induced diabetes. Immunoreactivity was detected by diaminobenzidine (brown) [n=5 per group; WT = wildtype, Con = nondiabetic controls, STZ = streptozotocin-induced diabetic mice, MD2KO = $MD2^{-/-}$ mice]. (**b**) Lysates prepared heart tissues were subjected to immunoblotting for macrophage marker CD68, and adhesion molecules intercellular cell adhesion molecule-1 (ICAM-1) and vascular cell adhesion molecule (VCAM-1). GAPDH was used as loading control [n = 6 or 7 per group].

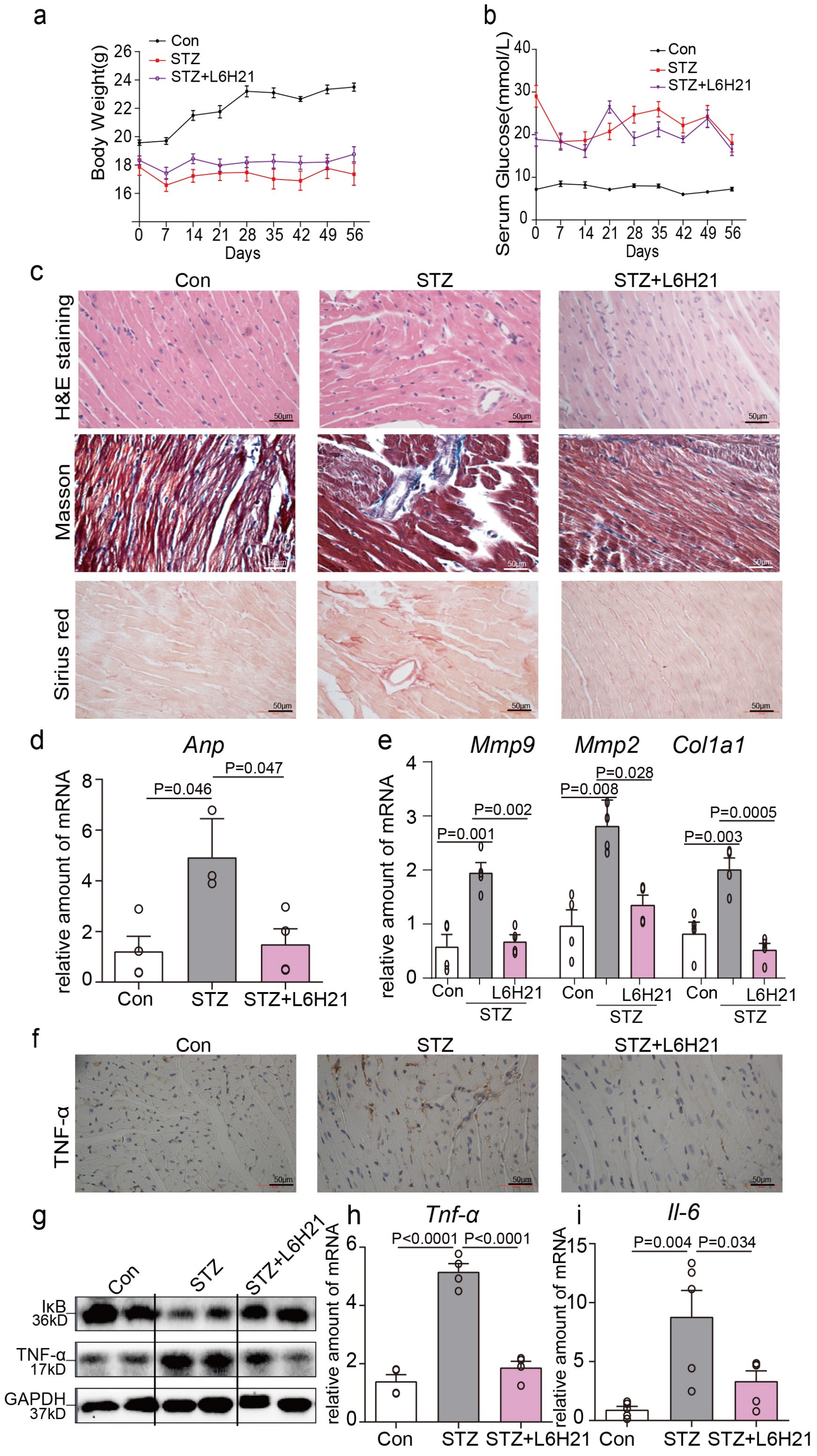

Supplementary Figure 8: MD2 inhibitor L6H21 protects against cardiac inflammatory injury in streptozotocin-induced diabetic mice. Male C57BL/6 mice were made diabetic by single

intraperitoneal injection of streptozotocin (STZ). Non-diabetic controls received citrate buffer only. Upon confirmation of hyperglycemia, diabetic mice were divided into two groups: STZ mice and STZ mice treated with small molecule inhibitor of MD2, L6H21. L6H21 was administered at 20 mg/kg/2d for 16 weeks. Untreated STZ mice received 1% CMC-Na vehicle [Con = non-diabetic controls, n=5; STZ = streptozotocin-induced diabetic mice, n=8; STZ+L6H21 = diabetic mice treated with L6H21, n=8]. (**a**, **b**) Body weights (a) and fasting blood glucose levels (b) were measured weekly for 16 weeks (Values are reported as Mean ± SEM). (**c**) Representative images (n=5 per group) of myocardial tissue stained with H&E (Upper panel), Masson's trichrome stain (middle panel), and Sirius red staining (Lower panel). (**d**) mRNA levels of *Anp* were determined by qPCR. Data was normalized to *Actb* [Mean ± SEM; n=4 per group]. (**e**) mRNA levels of tissue remodeling markers *Mmp9*, *Mmp2* and *Col1a1* [Mean ± SEM; n=4 per group]. (**f**) Representative immunohistochemical staining of tumor necrosis factor-α (TNF-α; brown) in cardiac tissues (n=5 per group). Slides were counterstained with hematoxylin. (**g**) Representative immunoblot image of inhibitor of κB (IκB) and TNF-α in mouse cardiac tissues (n=5 per group). GAPDH was used as loading control. (**h, i**) mRNA levels of *Tnfa* and *Il6* in heart tissues of diabetic mice treated with MD2 inhibitor [Mean ± SEM; n=4 per group in panel h; n=5 per group in panel i]. P-values by one-way ANOVA in d, e, h, i followed by Tukey's post hoc test are indicated.

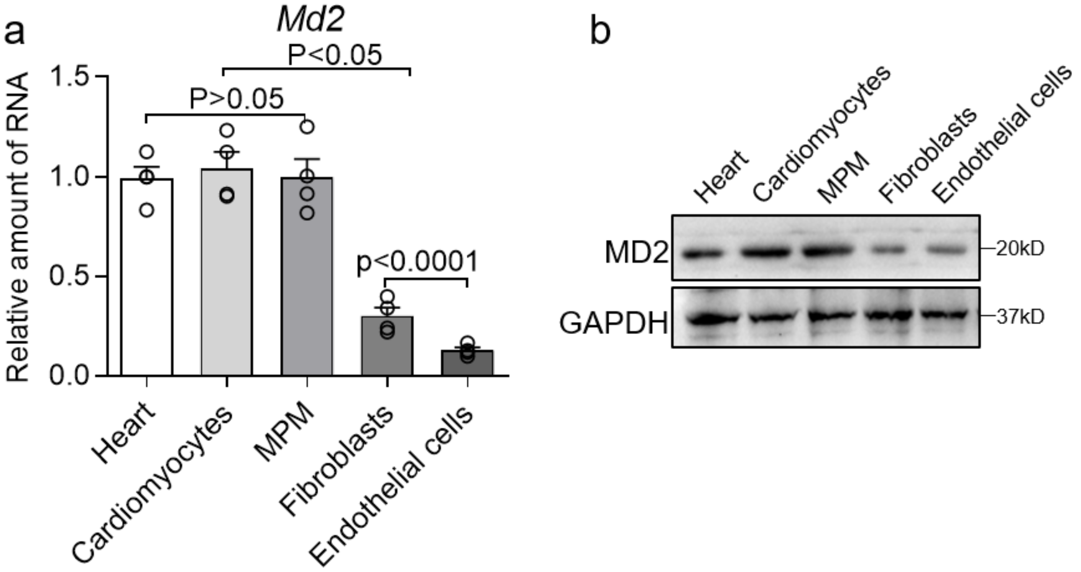

Supplementary Figure 9: MD2 expression in cardiac cells. *Md2* expression level in cardiac cells was determined by qPCR (**a**) and immunoblotting (**b**). Primary cardiomyocytes, endothelial cells, and fibroblasts were isolated from heart tissues of C57BL/6 mice. Mouse peritoneal macrophages (MPMs) were also analyzed. Heart tissue RNA and protein lysates were used as control. mRNA levels were normalized to *Actb* [Mean ± SEM; n =4 examinations]. GAPDH was also used as loading control for immunoblotting. P-values by one-way ANOVA in a followed by Tukey's post hoc test are indicated.

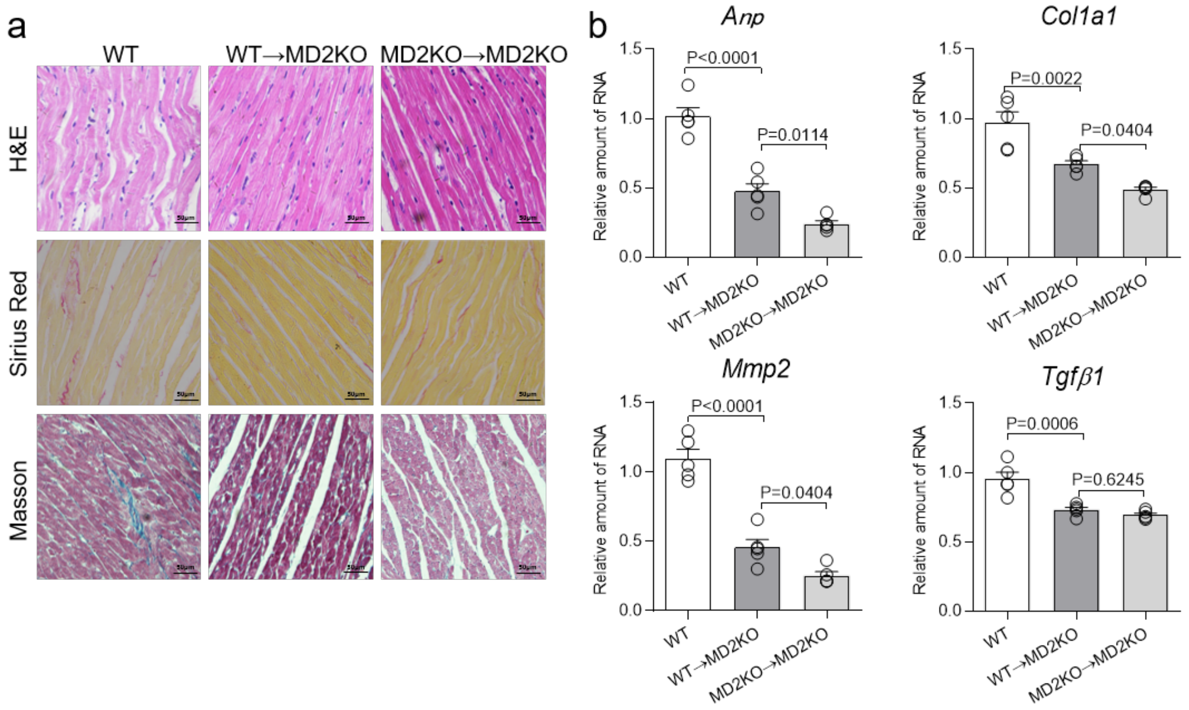

Supplementary Figure 10: Bone marrow reconstitution reveals important contribution of cardiac cell- and macrophage-derived MD2 in diabetes-induced cardiac injury. *Md2*-/- mice (MD2KO) were given acidified water containing neomycin and polymyxin B sulphate. One day prior to transplantation, mice were subjected to total body irradiation (6 Gy). Bone marrow cells prepared from wildtype (WT) or MD2KO mice were prepared and injected intravenously at 5 x10^6 cells. Tail clip samples and peritoneal macrophages were used for genotyping and confirmation of reconstitution. Mice were then made diabetic by streptozotocin (STZ). Heart tissues were harvested 12 weeks following the onset of diabetes. (**a**) Representative staining images of heart tissues showing H&E (upper panel), Sirius Red (middle panel), and Masson's Trichrome (lower panel) [n=5 per group]. (**b**) mRNA levels of *Anp*, *Col1a1*, *Mmp2*, and *Tgfb1* in heart tissues as determined by qPCR. mRNA data was normalized to *Actb* [Mean ± SEM; n=5 per group]. P-values by one-way ANOVA in b followed by Tukey's post hoc test are indicated.

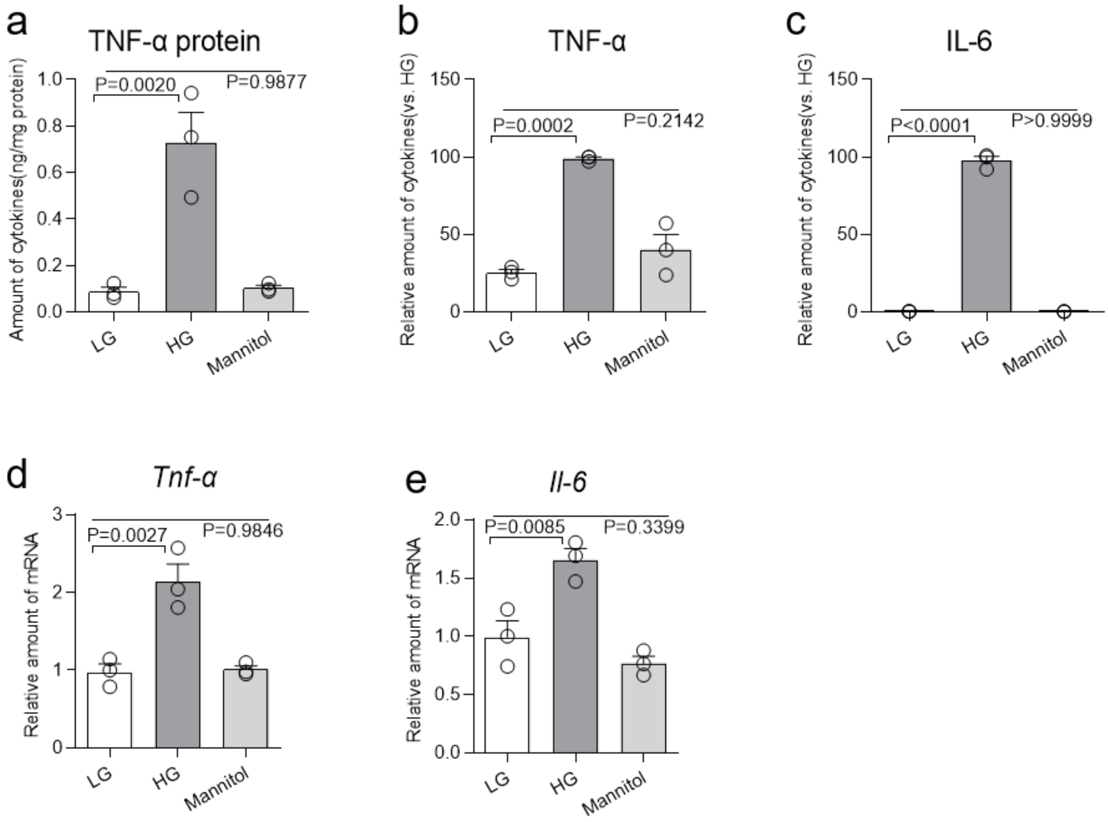

Supplementary Figure 11: Mannitol does not induce pro-inflammatory cytokines in H9C2 cells and macrophages. H9C2 cells and mouse peritoneal macrophages (MPMs) were cultured in normal/low glucose (5.5 mM), high glucose (HG, 33 mM) or osmotic control mannitol (33 mM). mRNA was isolated after 12-hour exposure and condition media after 24 hours. (**a**) Levels of TNF-α in H9C2 condition media was determined by ELISA [Mean ± SEM; n=3]. (**b**, **c**) Levels of TNF-α (b) and IL-6 (c) in condition media from MPMs was determined following 24 h of treatment [Mean ± SEM; n=3]. (**D**, **E**) mRNA levels of *Tnfa* (d) and *Il6* (e) in MPMs exposed to glucose or mannitol for 12 hours [Mean ± SEM; n = 3]. P-values by one-way ANOVA in a, b, c, d, e followed by Tukey's post hoc test are indicated.

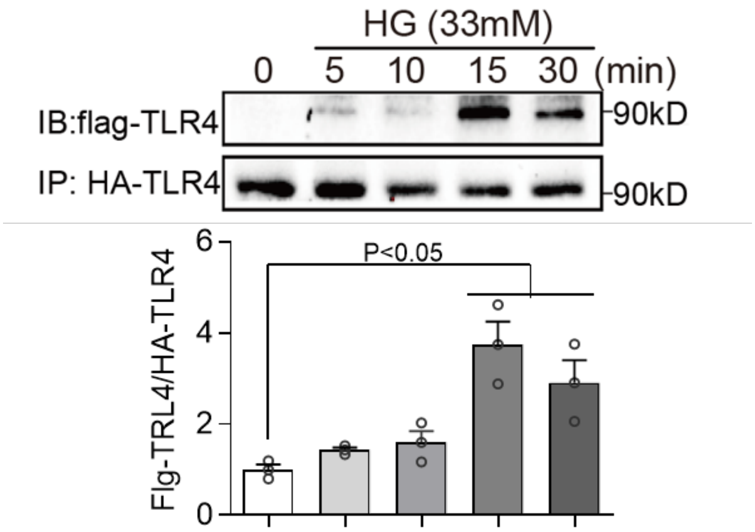

Supplementary Figure 12: HG induces TLR4 dimerization in H9C2 cells. HA-and FLAG-tagged rat *Tlr4* plasmids were constructed and sequenced by Shanghai GeneChem Co. Ltd. (Shanghai, China). H9C2 cells were transfected with both plasmids using Lipofectamine 3000 (Invitrogen). After 12 h, cells were incubated with HG (33 mM glucose) for indicated time points. Cell extracts were precipitated using HA antibody and immunoblotting was performed using FLAG antibody. Representative immunoprecipitation blots of FLAG-HA complex showing HG-induced dimerization of TLR4 in H9C2 cells [IP = precipitating antibody, IB = immunoblot antibody; n=3]. P-values by one-way ANOVA in lower panel followed by Tukey's post hoc test are indicated.

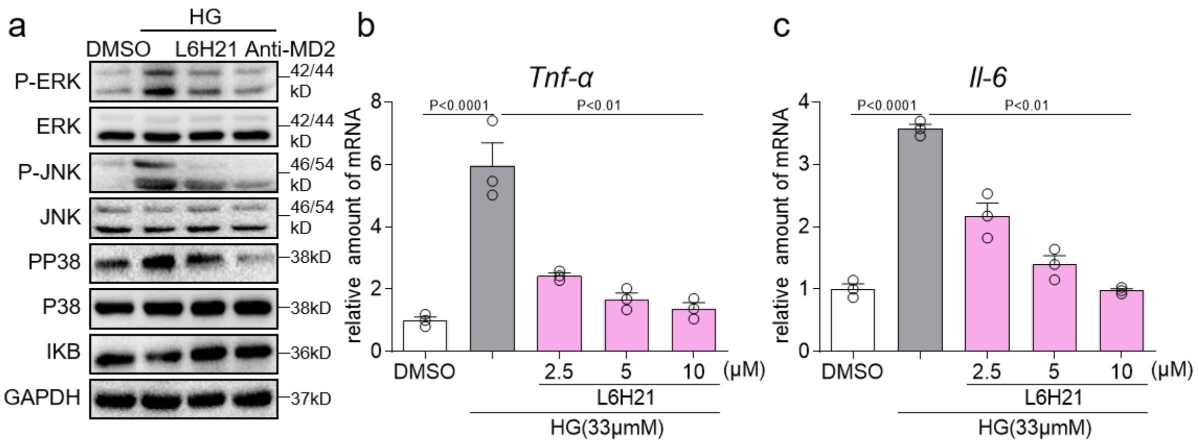

Supplementary Figure 13: MD2 inhibition blocked HG-induced inflammatory responses in H9C2 cells. (**a**) H9C2 cells were pretreated with 10 μM L6H21 or 100 ng/mL MD2 neutralizing antibody (anti-MD2) for 1 h. Cells were then exposed to HG (33 mM D-glucose) for 30 min. Representative Western blot image showing phosphorylated mitogen-activated protein kinase pathway proteins (p-ERK, p-JNK, and p-P38) and IκB. GAPDH was used as loading control [n=3 examinations]. (**b**, **c**) H9C2 cells were pretreated with L6H21 (2.5, 5, or 10 μM) or DMSO vehicle for 1 h. Cells were then stimulated with HG (33 mM glucose) for 6 h. mRNA levels of *Tnfa* and *Il6* were detected by qPCR [normalized to *Actb* mRNA; Mean ± SEM; n=3 examinations]. P-values by one-way ANOVA in **b, c** followed by Tukey's post hoc test are indicated.

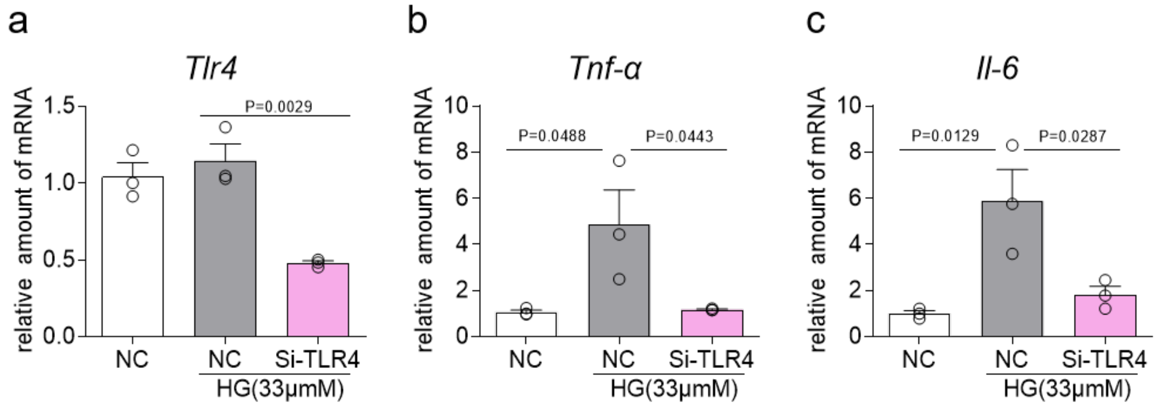

Supplementary Figure 14: TLR4 knockdown prevents HG-induced inflammatory cytokine production. H9C2 cells were transfected with *Tlr4* targeting siRNA (siTLR4) or negative control scramble sequences (NC). Transfected cells were then treated with HG (33 mM D-glucose) for 12 hours. (**a**) mRNA levels of *Tlr4* in cells transfected with *Tlr4* siRNA [n=3 examinations; Mean ± SEM]. (**b**) mRNA levels of *Tnfa* and *Il6* in H9C2 cells transfected with *Tlr4* siRNA [n=3 examinations; Mean ± SEM]. P-values by one-way ANOVA in **a, b, c** followed by Tukey's post hoc test are indicated.

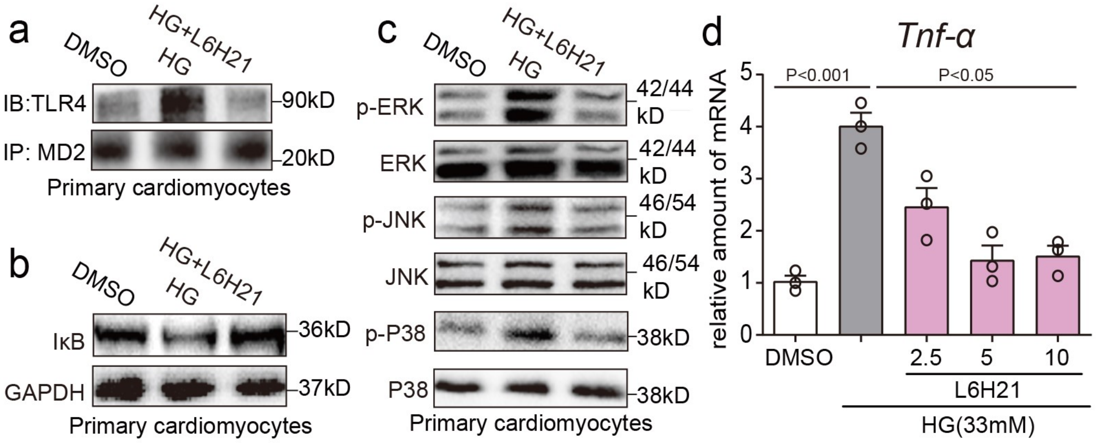

Supplementary Figure 15: L6H21 inhibits HG-induced inflammatory signaling in rat primary cardiomyocytes. Primary cardiomyocytes isolated from Sprague Dawley rats were pretreated with L6H21 or DMSO control (Ctrl) for 1 h and then exposed to HG (33 mM glucose) for different time points. (**a**) Representative immunoblot showing MD2-TLR4 complex formation in primary cardiomyocytes in response to HG. Proteins were immunoprecipitated using MD2 antibody (IP) and TLR4 (IB) was detected. L6H21 pretreatment was carried out at 10 μM. Cells were then exposed to HG for 5 min [n=3 examinations]. (**b**, **c**) Representative Western blot analysis of IκB and phosphorylated mitogen-activated protein kinase proteins (p-ERK, p-JNK, and p-P38). L6H21 pretreatment was carried out at 10 μM. Cells were then exposed to HG for 30 min. Total proteins and GAPDH were used as controls [n=3 examinations]. (**d**) mRNA levels of *Tnfa* in rat primary cardiomyocytes after 6 h HG exposure. L6H21 pretreatment was carried out at indicated concentrations (μM) [Mean ± SEM; n = 3 examinations]. P-values by one-way ANOVA in d followed by Tukey's post hoc test are indicated.

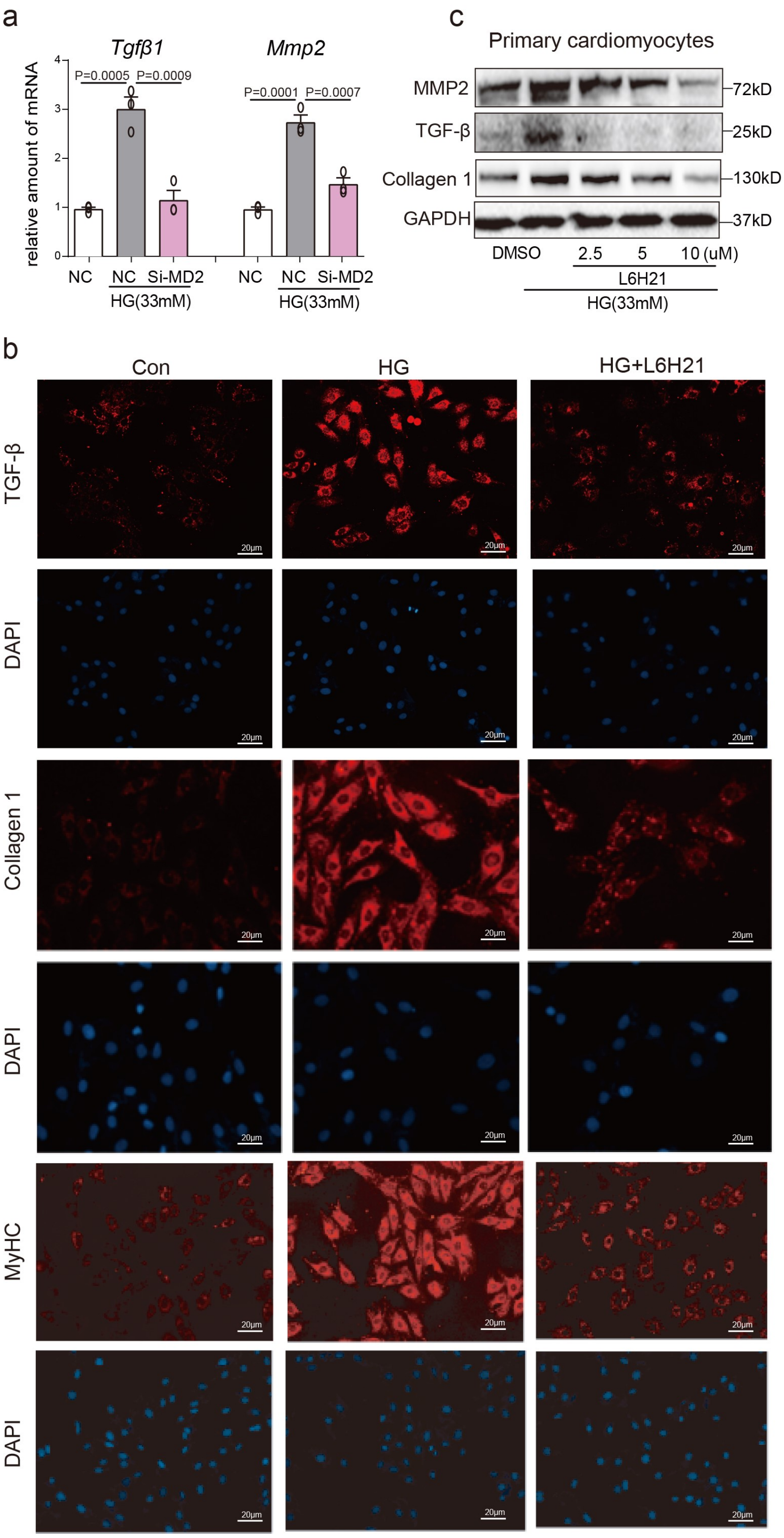

Supplementary Figure 16: MD2 deficiency reduces HG-induced tissue remodeling markers. (**a**) H9C2 cells were transfected with *Md2* siRNA or negative control sequences (NC). Transfected

cells were exposed to HG (33 mM glucose) for 24 h and RNA was isolated. Levels of *Tgfb1* and *Mmp2* were determined by qPCR Mean ± SEM; n = 3 examinations]. (**b**) H9C2 cells were pretreated with 10 μM L6H21 for 1 h and then exposed to HG (33 mM glucose) for 24 h. Cells were then stained for TGFβ1 (red), collagen I (red), and myosin heavy chain (MyHC) (red). DAPI (blue) was used to counterstain [n=3]. (**c**) Rat primary cardiomyocytes were pretreated with L6H21 at 2.5, 5.0, or 10 μM for 1 h and then exposed to HG (33 mM glucose) for 24 h. Protein levels of TGFβ1, MMP2, and collagen 1 were detected by immunoblotting. GAPDH was used as loading control [n=3 examinations]. P-values by one-way ANOVA in a followed by Tukey's post hoc test are indicated.

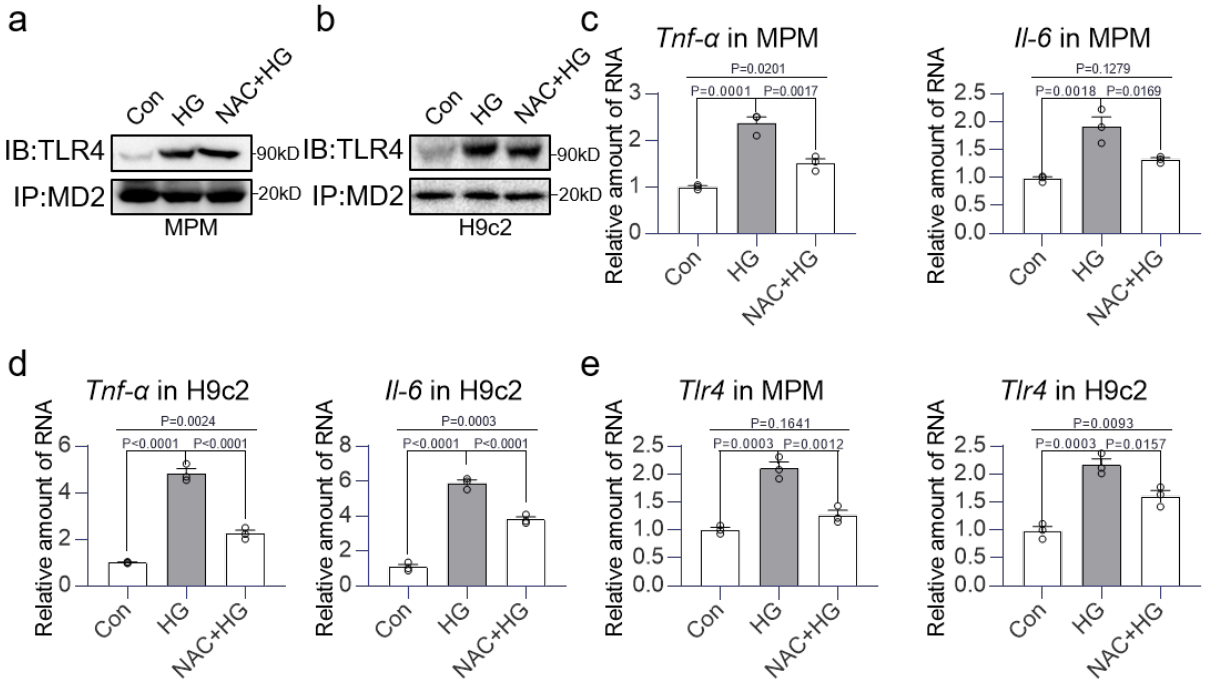

Reviewer Figure 17: Intracellular HG increases the expression of TLR4 via ROS but is not involved in MD2-TLR4 complex formation and TLR4 activation. (**a**) MD2-TLR4 interaction in mouse primary macrophages (MPM) exposed to HG with or without ROS scavenger N-acetyl cysteine (NAC; 5 mM) pretreatment. Cells were exposed to 33 mM HG for 15 minutes following a 1-hour pretreatment with 5 mM NAC. MD2 was immunoprecipitated (IP) and interaction with TLR4 was determined by immunoblotting (IB). Representative blots are from 3 independent experiments. (**b**) MD2-TLR4 interaction in H9C2 cells. Cells were treated as indicated for Panel a. Representative blots are from 3 independent experiments. (**c**) Mouse primary macrophages were exposed to 33 mM glucose (HG) for 12 hours, with or without 1h pretreatment with NAC (5 mM). mRNA levels of *Tnfa* and *Il6* were measured by real-time qPCR [mRNA data normalized to *Actb*; Mean ± SEM; n=3 examinations]. (**d**) *Tnfa* and *Il6* mRNA levels in H9C2 cells. Cells were treated as indicated for Panel c [Mean ± SEM; n=3 examinations]. (**e**) *Tlr4* expression in mouse primary macrophages and H9C2 cells following exposure to HG. Cells were treated as indicated for Panel c [mRNA data normalized to *Actb*; Mean ± SEM; n=3 examinations]. P-values by one-way ANOVA in c, d, e followed by Tukey's post hoc test are indicated.

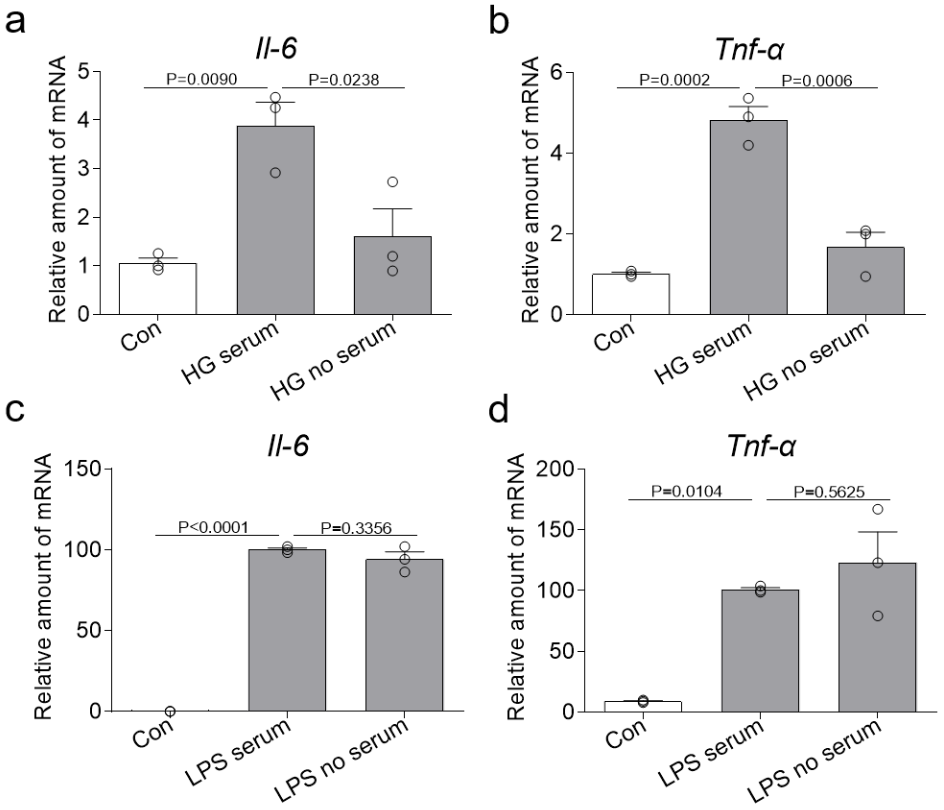

Supplementary Figure 18: HG-induced cytokine production is dependent on the presence of serum. (**a**, **b**) H9C2 cells were exposed to HG (33 mM glucose) for 6 h, in the presence or absence of heat-inactivated serum. Levels of *Il6* and *Tnfa* mRNA were determined [Mean ± SEM; n=3 examinations]. (**c**, **d**) H9C2 cells were exposed to 0.5 μg/mL lipopolysaccharide (LPS) for 6 h, in presence or absence of heat-inactivated serum. *Il6* and *Tnfa* mRNA levels were then determined [Mean ± SEM; n=3 examinations]. P-values by one-way ANOVA in a, b, c, d followed by Tukey's post hoc test are indicated.

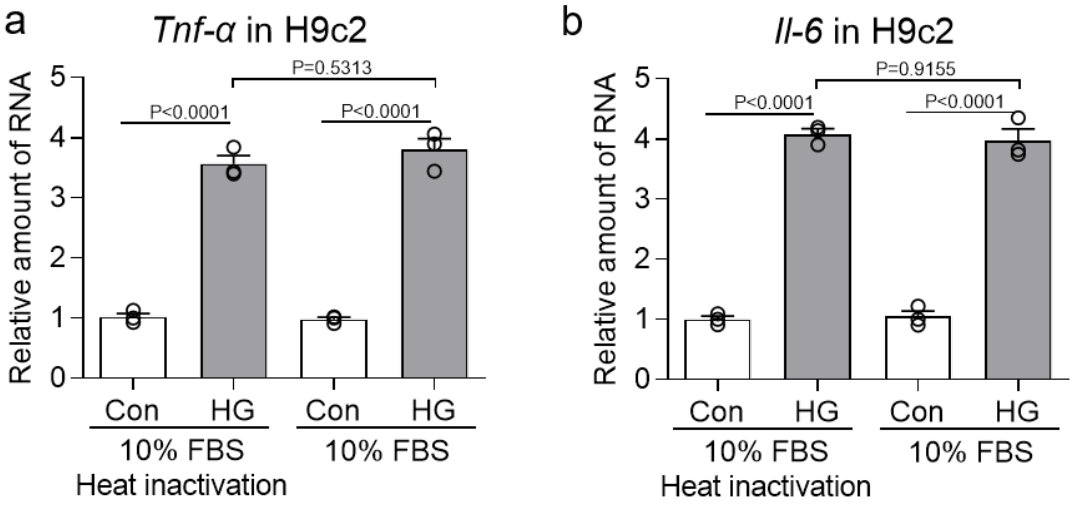

Supplementary Figure 19: Effect of heat-inactivation on HG-induced inflammatory cytokine production in H9C2 cells. (**a**, **b**) H9C2 cells were cultured in media containing 10% heat-inactivated fetal bovine serum or serum that did not undergo heat-inactivation for 10 days. Cells were then exposed to HG (33mM glucose) for 24 h. mRNA levels of *Tnfa* (a) and *Il6* (b) were determined [Mean ± SEM; n = 3 examinations]. P-values by one-way ANOVA in a, b followed by Tukey's post hoc test are indicated.

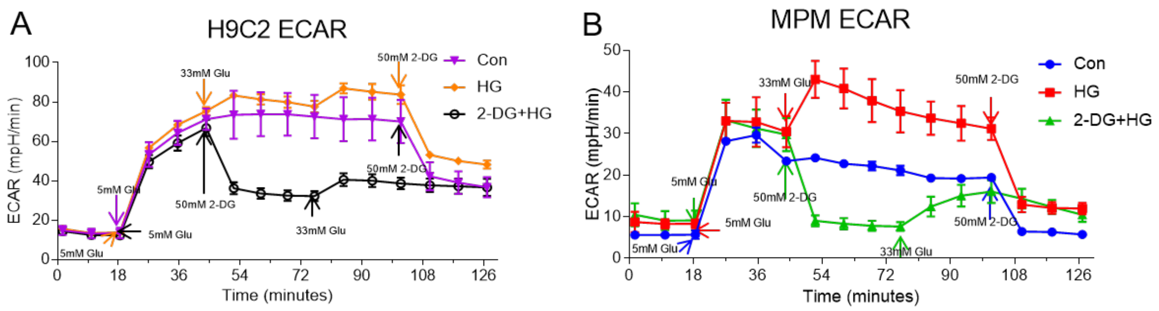

Supplementary Figure 20: Effect of high glucose on glycolytic function of cardiomyocytes and macrophages. Glycolytic function of cells was determined by Agilent Seahorse XF Glycolysis Stress Test Kit. Extracellular acidification rate (ECAR; mpH/min/µg protein) in H9C2 (**a**, 88000 cells per group) and MPMs (**b**, 22000 cells per group) exposed to HG (33 mM glucose). Sequential addition of glucose (Glu) and 2-deoxy-glucose (2-DG) is indicated by arrows. No changes in glycosis was observed in H9C2 cells exposed to HG compared to cells cultured in 5 mM glucose. Glycosis was increased in MPMs exposed to HG [n=3 repeats in Con and HG groups; n=4 repeats in DG+HG group; Values are reported as Mean ± SD].

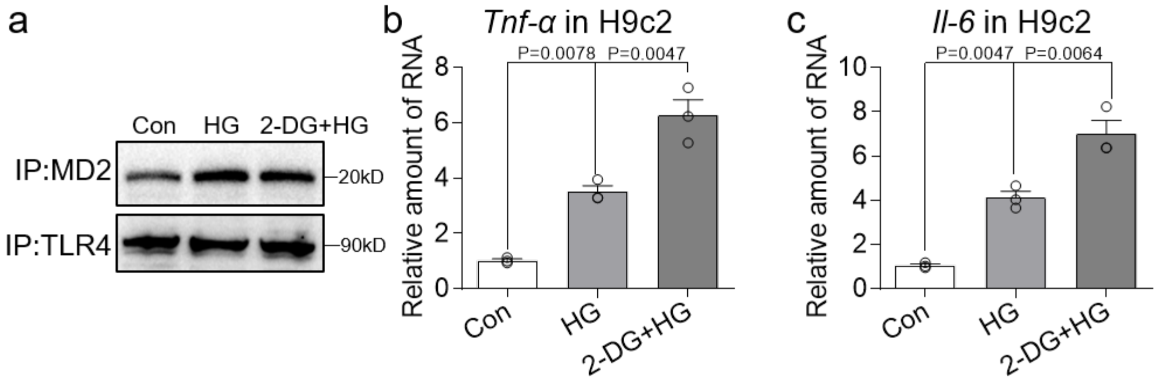

Supplementary Figure 21: Effect of glycolysis inhibitor on HG-induced MD2-TLR4 complex formation and inflammation. (**a**) H9C2 cells were pretreated with 50 mM 2-DG for 30 min and then exposed to HG (33mM glucose) for 15 min. Proteins were immunoprecipitated with TLR4 antibody (IP) and levels of MD2 were determined by immunoblotting (IB) [n = 3 examinations]. (**b**, **c**) H9C2 cells were pretreated with 50 mM 2-DG for 30 min and then exposed to HG (33mM glucose) for 12h. mRNA levels of *Tnfa* (b) and *Il6* (c) were determined by real-time qPCR assay. Glycolysis inhibitor 2-DG dose not suppress HG-increased mRNA levels of *Tnfa* and *Il6* in H9C2 cells. In contrast, glycolysis inhibitor 2-DG aggravates HG-induced *Tnfa* and *Il6* expression, possibly due to the decreased glucose consuming and increased extracellular glucose concentration. [Mean ± SEM; n=3 examinations]. P-values by one-way ANOVA in b, c followed by Tukey's post hoc test are indicated.

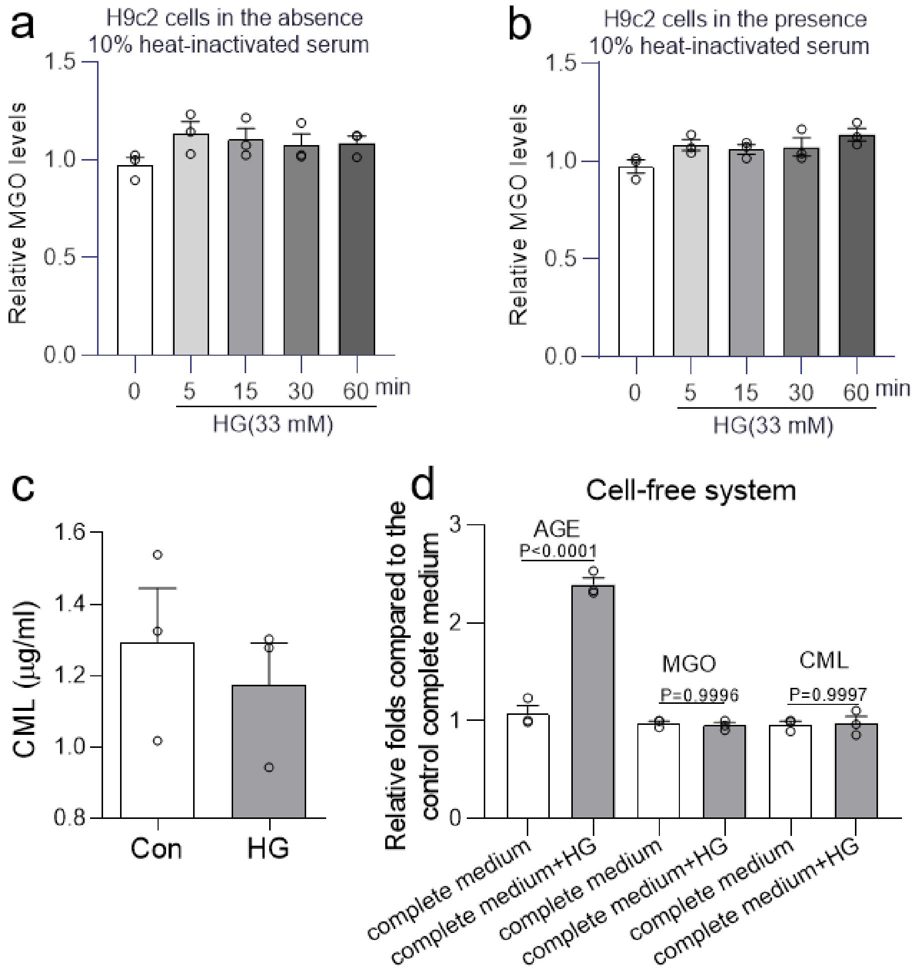

Supplementary Figure 22: Levels of methylglyoxal and Nε-carboxymethyl-lysine in H9C2 cells exposed to high levels of glucose. (**a**, **b**) H9C2 cells were exposed to 33 mM glucose (HG) for different time periods, either in the absence (a) or presence of 10% heat-inactivated serum (b). Levels of methylglyoxal (MGO) were measured by two-photon fluorophore conjugated to o-phenylenediamine which contains a MGO recognition site [n=3 examinations; Values are reported as Mean ± SEM]. (**c**) H9C2 cells were exposed to 33 mM glucose for 15 minutes in the presence of 10% heat-inactivated serum and levels of CML were measured by ELISA [n=3; Values are reported as Mean ± SEM]. (**d**) HG incubation in cell-free medium do not produce MGO and CML. Complete growth media containing serum was incubated with 33 mM glucose (HG) for 15 minutes. Levels of AGE-, MGO-, and CML-modified proteins were measured by the methods described in Methods section, respectively [n=3 examinations; Values are reported as Mean ± SEM]. P-values by unpaired t test are indicated in d.

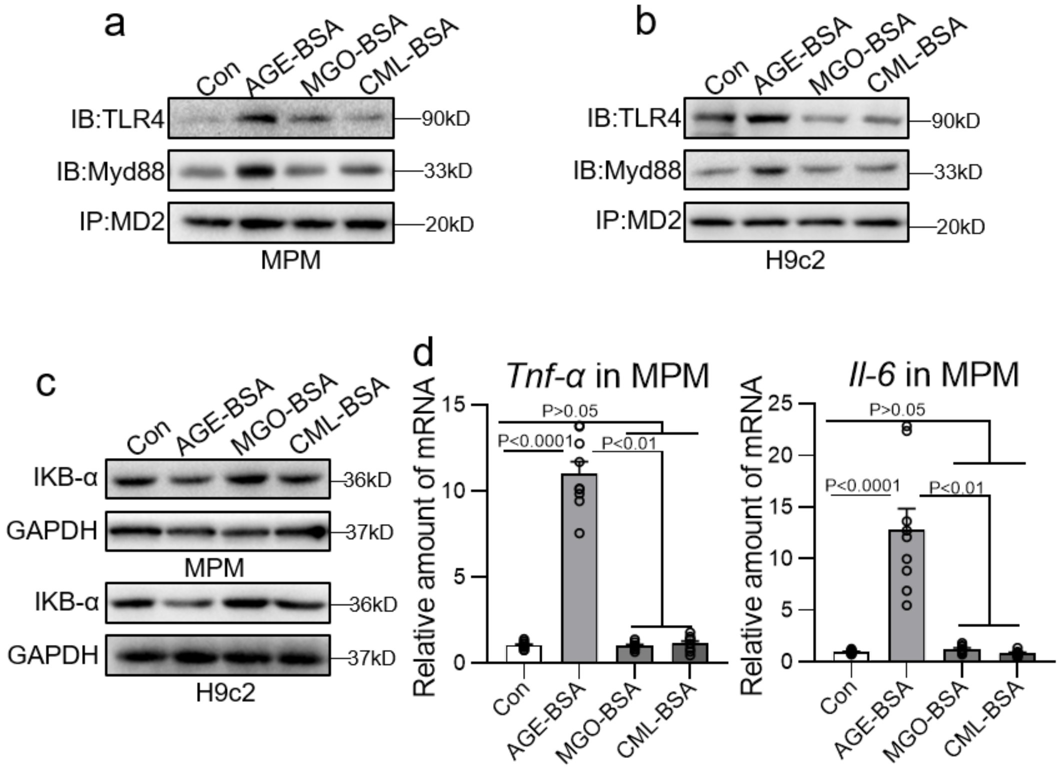

Supplementary Figure 23: AGE-BSA but not MGO-BSA or CML-BSA activates MD2/TLR4 inflammation. (**a**) Mouse primary macrophages were exposed to 30 μg/mL AGE-, MGO-, or CML-modified BSA for 15 minutes. Proteins were isolated, immunoprecipitated by MD2 antibody, and interaction between MD2-TLR4-MyD88 were assessed by immunoblotting. Cells in control group were exposed to 30 μg/mL BSA protein. Representative blots were shown from three independent experiments. (**b**) MD2-TLR4-MyD88 interaction in H9C2 cells. H9C2 cells were treated as indicated for Panel a. (**c**) Primary macrophages or H9C2 cellls were exposed to 30 μg/mL AGE-BSA, MGO-BSA, or CML-BSA for 1 hour. Cell lysates were probed for level of inhibitor of κB (IκBα). GAPDH was used as loading control. Cells in control group were exposed to 30 μg/mL BSA protein. Representative blots were shown from three independent experiments. (**d**) Real-time qPCR assay shows the mRNA levels of *Tnfa* and *Il6* in primary macrophages exposed to 30 μg/mL AGE-BSA, MGO-BSA, or CML-BSA for 12 hours [Con = 30 μg/mL BSA; n=9 repeats; Values are reported as Mean ± SEM]. P-values by one-way ANOVA in d followed by Tukey's post hoc test are indicated.

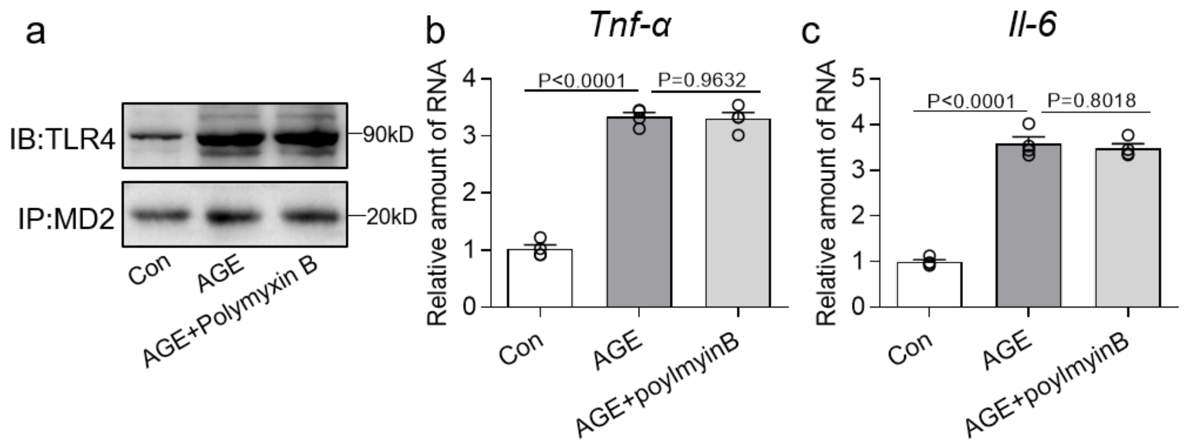

Supplementary Figure 24: MD2-binding polymyxin B does not alter AGE-induced inflammatory responses in H9C2 cells. (**a**) H9C2 cells were pretreated with 30 µg/mL polymyxin B for 1 hour and then exposed to 30 µg/mL AGE-BSA (AGE). AGE-BSA (with or without polymyxin B pretreatment) were carried out for 15 minutes. Protein samples were immunoprecipitated using MD2 antibody (IP) and levels of TLR4 were determined by immunoblotting (IB). Representative blots are from 3 independent experiments. (**b**, **c**) H9C2 cells were exposed to 30 µg/mL AGE-BSA for 24 hours, with or without pretreatment with 30 µg/mL polymyxin B for 1 hour. Levels of *Tnfa* (b) and *Il6* (c) mRNA were measured. Data was normalized to *Actb* [n=4 examinations; Values are reported as Mean ± SEM]. P-values by one-way ANOVA in b, c followed by Tukey's post hoc test are indicated.

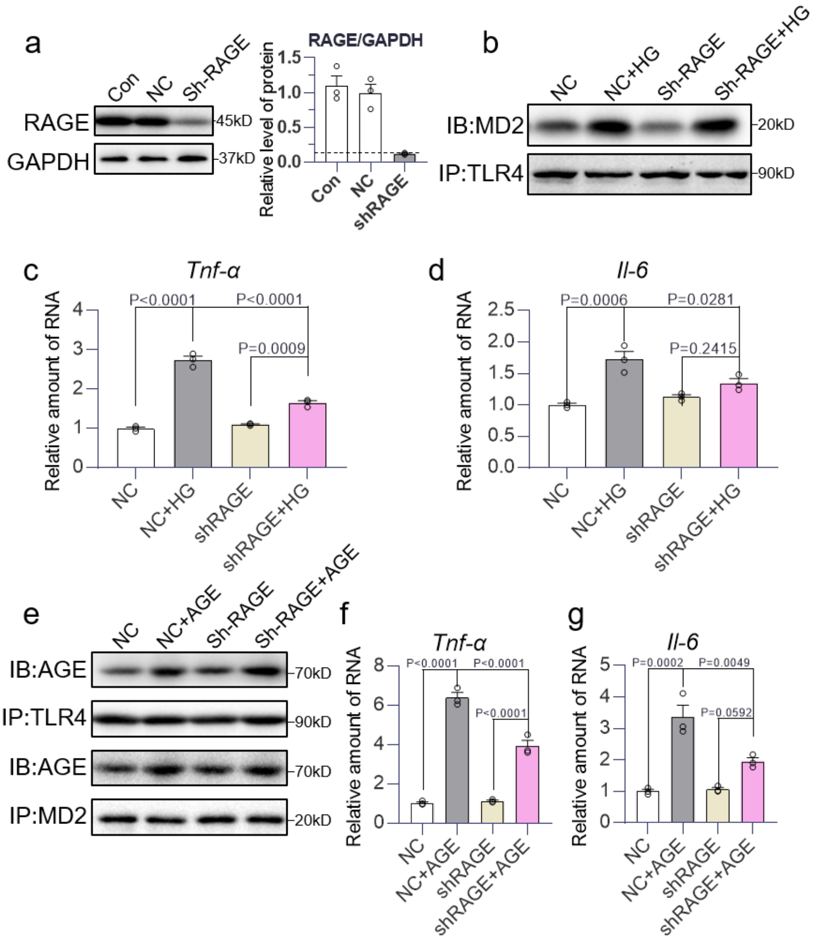

Supplementary Figure 25: RAGE knockdown does not alter HG- or AGEs-induced MD2-TLR4 activation in H9C2 cells. (**a**) RAGE in H9C2 cells was knocked down by shRNA (sh-RAGE) transfection. Control cells were transfected with negative control (NC) plasmids. Representative Western blot showing RAGE protein levels following transfection. GAPDH was used as loading control [n=3; Con = untransfected cells, NC = negative control plasmid, Sh-RAGE = RAGE shRNA plasmid]. The column figure shows a silence approximately 90% of RAGE expression. (**b**) H9C2 cells transfected with RAGE shRNA were exposed to HG (33 mM glucose) for 5 min. Proteins were immunoprecipitated using TLR4 antibody (IP) and levels of MD2 were determined by immunoblotting (IB) [n=3]. (**c**, **d**) mRNA levels of *Tnfa* (c) and *Il6* (d) in H9C2 cells were determined following RAGE shRNA transfection and exposure to HG (33 mM glucose). HG exposure was carried out for 6 h [Mean ± SEM; n=3 examinations]. (**e**) RAGE shRNA transfected H9C2 cells were exposed to HG (33 mM glucose) for 5 min. Interactions between AGE-MD2 and AGE-TLR4 were determined by co-immunoprecipitation [IP = precipitating antibody, IB = immunoblot antibody; n=3]. (**f**, **g**) H9C2 cells transfected with RAGE shRNA were exposed to 33 μg/mL AGE-BSA (AGE) for 6 h. mRNA levels of *Tnf1* (f) and *Il6* (g) were measured [Mean ± SEM; n=3 examinations]. P-values by one-way ANOVA in c, d, f, g followed by Tukey's post hoc test are indicated.

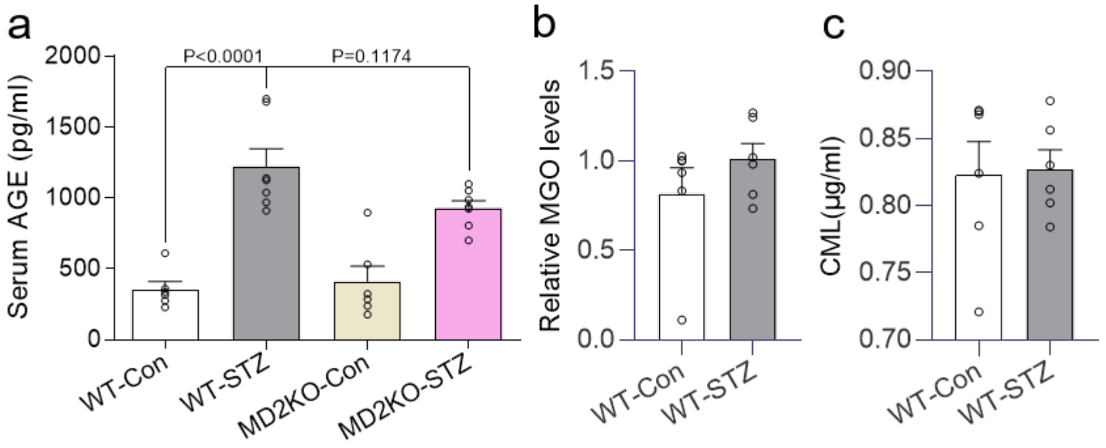

Supplementary Figure 26: AGE products in mouse model of type 1 diabetes. (**a**) Levels of AGE products in serum samples obtained from diabetic mice were determined by ELISA. Samples were obtained at week 16 following the onset of diabetes [Mean ± SEM; n=6 in WT-Con and MD2KO-Con group; n=7 in WT-STZ and MD2KO-STZ group]. (**b**, **c**) Levels of methylglyoxal (MGO, b) and Nε-carboxymethyl-lysine (CML, c) in heart tissues of non-diabetic control mice and streptozotocin-induced diabetic mice were measured by the methods described in Methods section, respectively [n=6; statistically non-significant; Values are reported as Mean ± SEM]. P-values by one-way ANOVA in a followed by Tukey's post hoc test are indicated.

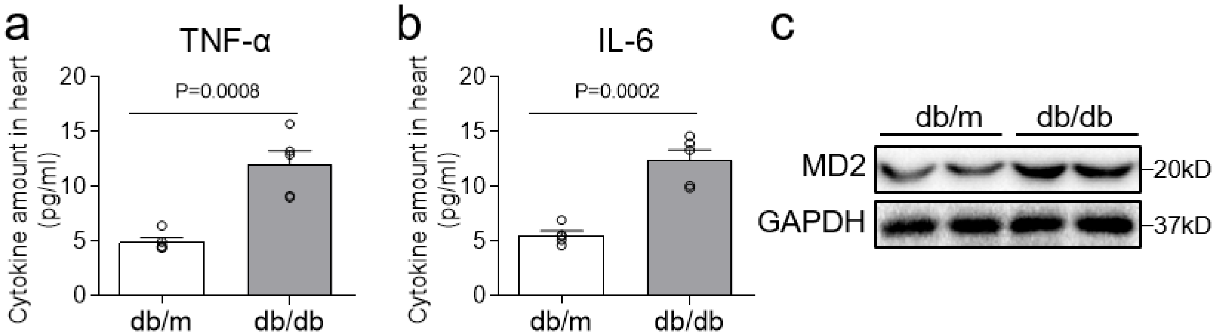

Supplementary Figure 27: MD2-TLR4 interaction and induction of inflammatory cytokines in model of type 2 diabetes. Male *db/db* (C57BLKS/J-leprdb/leprdb) mice were used to model type 2 diabetes. Male *db/m* littermates were used as controls. Mice were maintained on standard rodent diet for 16 weeks. (**a**, **b**) Heart tissues were harvested from mice and levels of TNF-α (a) and IL-6 (b) were measured by ELISA [Mean ± SEM; n=5 examinations]. (**c**) Representative Western blot analysis of MD2 protein in heart tissue of *db/db* mice. GAPDH was used as loading control [n=5 per group; two samples per group shown]. P-values by unpaired t test are indicated in a, b.

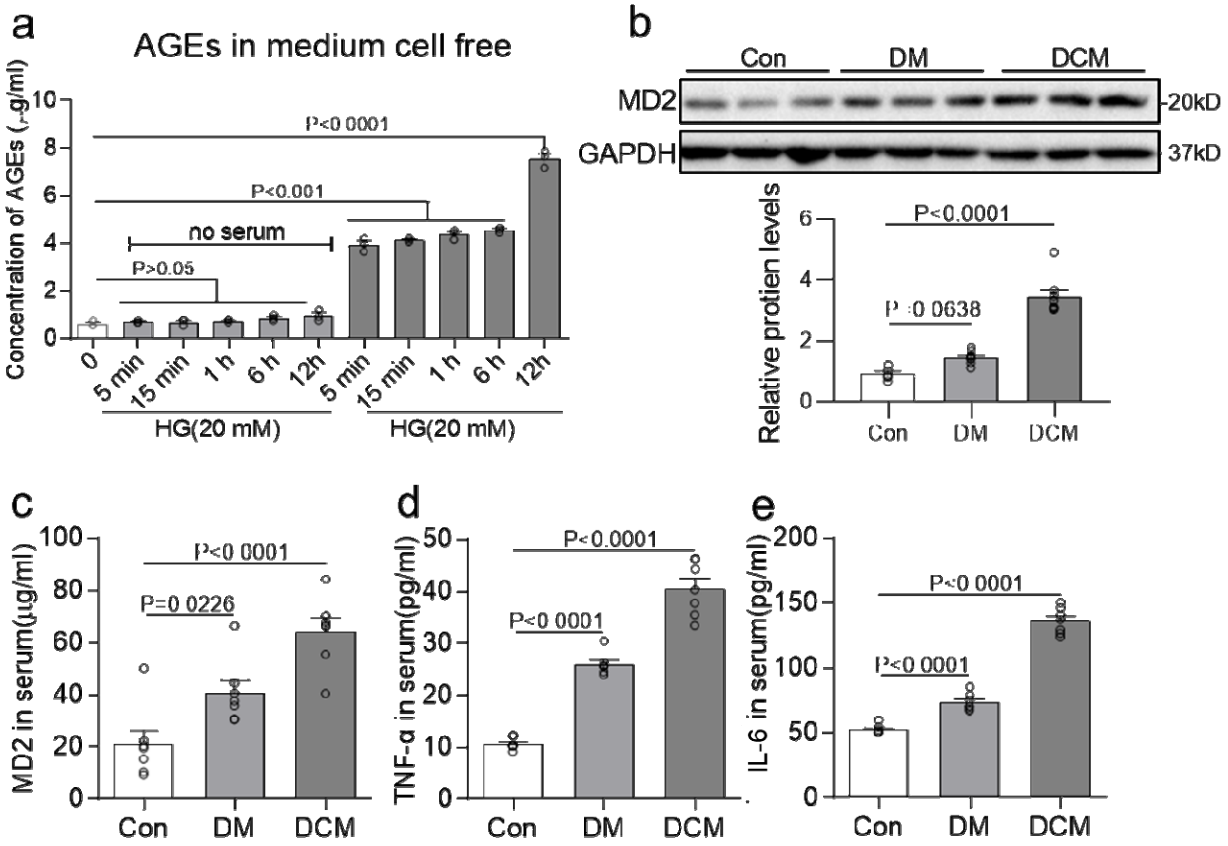

Supplementary Figure 28: AGE formation and induction of inflammatory cytokines in human diabetic subjects. (**a**) Serum from healthy volunteers was incubated with HG (20 mM glucose) for indicated time points. Levels of AGE products were measured by ELISA [no serum = PBS; Mean ± SEM; n=3 examinations]. (**b**) Western blot analysis of MD2 in peripheral blood mononuclear cells isolated from blood samples of healthy subjects (Con), diabetic subjects without cardiomyopathy (DM), and diabetic subjects with cardiomyopathy (DCM). GAPDH was used as loading control [two samples per group shown in immunoblots [Mean ± SEM; n=7 per group; three samples per group shown]. (**c-e**) Serum samples were obtained from healthy volunteers (non-diabetic control), diabetic subjects without cardiomyopathy (DM), and diabetic subjects with cardiomyopathy (DCM). Levels MD2 (c), TNF-α (d), and IL-6 (e) in human serum samples [Mean ± SEM; n=7 per group]. P-values by one-way ANOVA in b, c, d, e followed by Tukey's post hoc test are indicated.

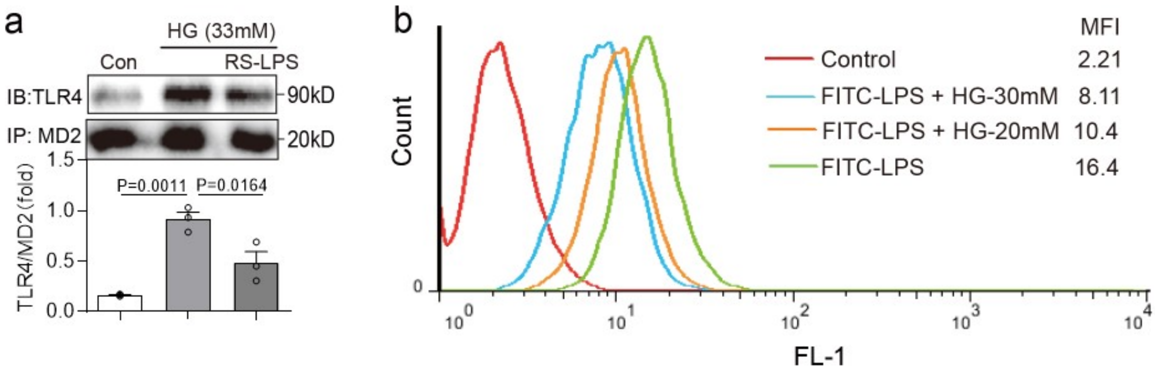

Supplementary Figure 29: Preliminary studies show that AGE products partially compete with LPS in MD2 interaction. (**a**) H9C2 cells were pretreated with 0.5 µg/mL LPS from *Rhodobacter sphaeroides* (RS-LPS) for 30 min. Cells were then exposed to HG (33 mM glucose) for 5 min. Proteins were immunoprecipitated with MD2 antibody (IP) and levels of TLR4 were determined by immunoblotting (IB). Lower panel showing quantification of MD2-TLR4 complex [Mean ± SEM; n=3]. (**b**) Cell surface interaction of LPS is inhibited by HG. H9C2 cells were cultured in 0.5 µg/mL FITC-labeled LPS (FITC-LPS) for 5 min, either in the presence of absence of HG (20 or 30 mM glucose). FITC-LPS interaction on cell surface was determined by flow cytometry and shown as mean fluorescence intensity (MFI) [n=3]. P-values by one-way ANOVA in a (lower panel) followed by Tukey's post hoc test are indicated.

Supplementary Table 1: Medical profile of human subjects.

Group	NO.	Age, years	Sex[1]	Blood glucose (mmol/l)	Diabetes duration, years	Complication[2]	LVEF, %[3]	Drugs[4]
Normal[5]	1	62	M	5.6	0	N	75.1	Non
	2	44	M	6.3	0	N	80.1	Non
	3	51	M	4.8	0	N	74.2	Non
	4	30	F	5.3	0	N	77.5	Non
	5	47	M	6	0	N	66.8	Non
	6	67	F	5.7	0	N	78.1	Non
	7	63	F	4.5	0	N	66.2	Non
	8	47	M	5.7	0	N	72.3	Non
Mean±SE		51.4±4.3	/	5.49±0.2	0	/	73.8±1.8	/
	1	35	M	9.2	0.08	N	71.1	Non
	2	49	M	13.7	0.17	N	67.9	Met, In
	3	47	M	9.7	0	N	72.7	Non
	4	38	M	15.3	0	N	67.4	Non
	5	60	F	10.8	1	N	72.3	Met
	6	53	F	9.8	0	***N***	69.8	Non
	7	63	F	10.6	0.17	N	74.3	Met
Mean±SE		54.1±4.3	/	11.3±0.87	0.20±0.14	/	70.79±0.97	/
DCM patient	1	65	M	11.8	20	Y	41	In, St
	2	58	M	13	10	Y	55.9	In, St
	3	60	M	14.4	8	Y	58.8	Met, Ac, Gl
	4	52	F	12.9	10	Y	66.5	In, Ac, Gl
	5	55	F	16.3	10	Y	53.4	In, Met
	6	81	M	13.1	10	Y	62	Ac
	7	72	M	12.8	2	Y	55.3	Ac
	8	67	M	10	10	Y	56	St
	9	57	F	17.8	20	Y	60	In, St
Mean±SE		63±3.1	/	13.57±0.78	11.1±1.89	/	56.54±2.3	/

[1] M, male; F, female.
[2] N=no, Y=yes; Micro- or macrovascular complications of diabetes (including nephropathy, neuropathy, retinopathy, peripheral vascular disease, ischemic heart disease, and stroke).
[3] left ventricular ejection fraction.
[4] In, insulin; St, statin; Met, Metformin; Ac, acarbose; Gl, gliclazide.
[5] Normal = Non-diabetic healthy

Supplementary Table 2: Primers for real-time qPCR.

Gene	Species	Forward	Reverse
Tnfa	Rat	TACTCCCAGGTTCTCTTCAAGG	GGAGGCTGACTTTCTCCTGGTA
Il6	Rat	GAGTTGTGCAATGGCAATTC	ACTCCAGAAGACCAGAGCAG
Il1b	Rat	GGGCCTCAAGGGGAAGAATC	ATGTCCCGACCATTGCTGTT
Tgfb1	Rat	GCAACAACGCAATCTATGAC	CCTGTATTCCGTCTCCTT
Mmp2	Rat	GTCCTGACCAAGGATATAGCC	AGACCCAGTACTCATTCCCTG
Md2	Rat	AGAGGCAACAGTGGATCTGC	GCGCTTCGGCAATTCTATGG
Tlr4	Rat	GAATGAGGACTGGGTGAGAAA	TCTGCTAAGAAGGCGATACAA
Actb	Rat	AAGTCCCTCACCCTCCCAAAAG	AAGCAATGCTGTCACCTTCCC
Mmp9	Mouse	GGACCCGAAGCGGACATTG	CGTCGTCGAAATGGGCATCT
Mmp2	Mouse	CCAGAAGGCGAACAGACTG	TGGGCCGGAGACCTAAAGAG
Col1a1	Mouse	TGGCCTTGGAGGAAACTTTG	CTTGGAAACCTTGTGGACCAG
Tnfa	Mouse	TGATCCGCGACGTGGAA	ACCGCCTGGAGTTCTGGAA
Il6	Mouse	GAGGATACCACTCCCAACAGACC	AAGTGCATCATCGTTGTTCATACA
Anp	Mouse	ACCTCCCGAAGCTACCTAAGA	CAACCTTTTCAACGGCTCCAA
Tgfb1	Mouse	TGACGTCACTGGAGTTGTACGG	GGTTCATGTCATGGATGGTGC
Actb	Mouse	CCGTGAAAAGATGACCCAGA	TACGACCAGAGGCATACAG
